# Current Trends in and Indications for Endoscopy-Assisted Breast Surgery for Breast Cancer: Results from a Six-Year Study Conducted by the Taiwan Endoscopic Breast Surgery Cooperative Group

**DOI:** 10.1371/journal.pone.0150310

**Published:** 2016-03-07

**Authors:** Hung-Wen Lai, Shou-Tung Chen, Dar-Ren Chen, Shu-Ling Chen, Tsai-Wang Chang, Shou-Jen Kuo, Yao-Lung Kuo, Chin-Sheng Hung

**Affiliations:** 1 Endoscopy & Oncoplastic Breast Surgery Center, Changhua Christian Hospital, No.135, Nanxiao Street, Changhua, 500 Taiwan (R.O.C.); 2 Division of General Surgery, Changhua Christian Hospital, No.135, Nanxiao Street, Changhua, 500 Taiwan (R.O.C.); 3 Comprehensive Breast Cancer Center, Department of Surgery, Changhua Christian Hospital, No.135, Nanxiao Street, Changhua, 500 Taiwan (R.O.C.); 4 School of Medicine, National Yang Ming University, No.155, Sec.2, Linong Street, Taipei, 112 Taiwan (R.O.C.); 5 Department of Surgery, National Cheng Kung University Hospital, College of Medicine, National Cheng Kung University, Tainan and Dou-Liou branch, N0. 138, Sheng Li Road, Tainan, 704 Taiwan (R.O.C.); 6 Division of General Surgery, Department of Surgery, Taipei Medical University Hospital, No. 252, Wu Hsing Street, Taipei, 110 Taiwan (R.O.C.); School of Medicine, Fu Jen Catholic University, TAIWAN

## Abstract

**Background:**

Endoscopy-assisted breast surgery (EABS) performed through minimal axillary and/or periareolar incisions is a possible alternative to open surgery for certain patients with breast cancer. In this study, we report the early results of an EABS program in Taiwan.

**Methods:**

The medical records of patients who underwent EABS for breast cancer during the period May 2009 to December 2014 were collected from the Taiwan Endoscopic Breast Surgery Cooperative Group database. Data on clinicopathologic characteristics, type of surgery, method of breast reconstruction, complications and recurrence were analyzed to determine the effectiveness and oncologic safety of EABS in Taiwan.

**Results:**

A total of 315 EABS procedures were performed in 292 patients with breast cancer, including 23 (7.8%) patients with bilateral disease. The number of breast cancer patients who underwent EABS increased initially from 2009 to 2012 and then stabilized during the period 2012–2014. The most commonly performed EABS was endoscopy-assisted total mastectomy (EATM) (85.4%) followed by endoscopy-assisted partial mastectomy (EAPM) (14.6%). Approximately 74% of the EATM procedures involved breast reconstruction, with the most common types of reconstruction being implant insertion and autologous pedicled TRAM flap surgery. During the six-year study period, there was an increasing trend in the performance of EABS for the management of breast cancer when total mastectomy was indicated. The positive surgical margin rate was 1.9%. Overall, the rate of complications associated with EABS was 15.2% and all were minor and wound-related. During a median follow-up of 26.8 (3.3–68.6) months, there were 3 (1%) cases of local recurrence, 1 (0.3%) case of distant metastasis and 1 (0.3%) death.

**Conclusion:**

The preliminary results from the EABS program in Taiwan show that EABS is a safe procedure and results in acceptable cosmetic outcome. These findings could help to promote this under-used surgical technique in the field of breast cancer.

## Introduction

Historically, modified radical mastectomy was the preferred method for treating operable breast cancer[1]. However, a number of advances in surgical techniques have been made over the past few decades and now breast-conserving surgery (BCS) is increasingly being used as treatment for breast cancer, especially in women with early stage disease[2, 3]. Sentinel lymph node biopsy (SLNB) is now performed in most patients thereby sparing the need for axillary lymph node dissection (ALND) in clinical node negative patients[4]. Another important advancement in the field of breast surgery has been the development of oncoplastic breast surgery, a breast-conserving technique that combines wide tumor excision with immediate partial breast reconstruction using either volume displacement or volume replacement techniques[5, 6]. Nonetheless, mastectomy is still indicated for some patients, especially for women with large tumors or multi-centric lesions[7]. Fortunately, recent advances in the field now allow for nipple sparing mastectomy (NSM) with immediate breast reconstruction (IBR) to be performed[8, 9], which results in much better cosmetic outcome and quality of life than conventional mastectomy[10].

Endoscopic (or laparoscopic) surgery, a technique that optimizes cosmetic outcome because it is performed through small wounds hidden in inconspicuous areas, is widely used in the gastrointestinal[11, 12], urologic[13], and thoracic surgical fields[14]. Endoscopy-assisted breast surgery (EABS), which is performed through minimal axillary and/or periareolar incisions, was initially developed to facilitate breast augmentation[15–17], but is now increasingly used to excise benign breast tumors[18–20], resect malignant breast tumors[21–24], and to assist in SLNB[25, 26].

EABS has been shown to be an effective breast-conserving technique for early breast cancer[22, 24, 27–29]. In addition, endoscopic approaches can be used to perform skin-sparing mastectomy (SSM) and NSM [23, 30] followed by IBR with implants[31–33] or autologous flaps[34, 35]. EABS is used as an alternative to conventional surgery in select patients with early stage breast cancer in a few Western countries[36] and in some Asian countries, such as Japan[19, 21, 23, 24, 28, 29, 31, 37, 38], China[30, 32], and Korea[22, 27]. However, the use of EABS in the management of breast cancer has yet to become a mainstream treatment modality mainly because there is an absence of randomized level I clinical evidence showing that EABS achieves oncologic outcomes equivalent to open surgery[36, 39].

In this study, we report the preliminary results of an EABS program in Taiwan. The study investigated the trends in and types of EABS performed and the oncologic outcomes in patients who underwent EABS for primary operable breast cancer at three major endoscopic breast surgery centers in Taiwan during the period 2009–2014.

## Materials and Methods

### Patients

The Taiwan Endoscopic Breast Surgery Cooperative Group (T-EBSCG) was established to monitor the effectiveness of and clinical outcome associated with EABS in Taiwan. The T-EBSCG comprises members from three major endoscopic breast surgery centers, namely Changhua Christian Hospital (CCH), a tertiary medical center located in central Taiwan, National Cheng-Kung University Hospital (NCKUH), a tertiary medical center located in southern Taiwan, and Taipei Medical University Hospital (TMUH), a tertiary medical center located in northern Taiwan. In this study, we collected clinicopathologic data from the T-EBSCG database on patients who underwent EABS for breast cancer during the period May 2009 to December 2014 at the three T-EBSCG-affiliated institutions. The data gathered from the database covered more than 90% of the endoscopic breast surgeries performed in Taiwan, and therefore can be interpreted as representing the status of EABS in Taiwan.

The data collected from the database included clinicopathologic characteristics of patients, type of mastectomy, method of breast reconstruction (implant or flap), whether the surgery was performed concomitantly with contralateral surgery, operative time, blood loss, hospital stay, complications, recurrence and survival status at last follow-up. All data were collected by chart review by a specially trained nurse and were confirmed by the principle investigator (HWL). The study was approved by the Institutional Review Board of the Changhua Christian Hospital (CCH IRB No.: 141224). Written informed consent to the use of clinical records was obtained from each participant. This current report includes photos of several patients, and they had agreed and signed the consent for publication of their pictures.

Pre-operative sonograms and mammograms were used to determine the eligibility of patients for EABS. Liver sonogram, chest X ray, and whole body bone scan were used to exclude the possibility of distant metastasis. Indications for EABS included early stage breast cancer (ductal carcinoma in situ (DCIS), stage I or II), a tumor size less than 3 cm for endoscopic-assisted partial mastectomy (EAPM) or no larger than 5 cm for endoscopic assisted total mastectomy (EATM), no evidence of multiple lymph node metastasis, and no evidence of skin or chest wall invasion. Patients for whom EABS was contraindicated included those with inflammatory breast cancer, breast cancer with chest wall or skin invasion, locally advanced breast cancer, breast cancer with extensive axillary lymph node metastasis (stage IIIB or later), and patients with severe co-morbid conditions, such as heart disease, renal failure, liver dysfunction, and poor performance status as assessed by the primary physicians. The inclusion and exclusion criteria were based on those reported previously[23, 24, 36].

For the perioperative safety evaluation, we analyzed operative time, blood loss, hospital stay, and postoperative complications, including wound healing, infection, seroma formation, nipple ischemia or necrosis, and implant or flap loss. For the oncological safety evaluation, we analyzed the rate of positive surgical margin involvement, locoregional recurrence, distant metastasis, disease-free survival (DFS) and breast cancer-specific survival (BCSS). Surgical margin involvement was defined as the presence of breast cancer cells located less than 1 mm from the peripheral margin of resected specimens. Surgical margins less than 1 mm from the superficial (away from skin flap) or deep (away from pectoralis major muscle) layer of the fascia, where the fibroglandular boundary of the skin and chest wall was located, were not regarded as margin involvement [40]. Locoregional recurrence was defined as the reappearance of cancer at the operative breast or axilla. Distant metastasis was defined as any recurrence in distant organs. DFS was defined as freedom from breast cancer recurrence or death, and BCSS was defined as freedom from breast cancer death. Total incidence of recurrence or death due to breast cancer was ascertained at the most recent follow-up, which ended on 31 March 2015.

### Endoscopic breast surgery technique

Details of the surgical technique for EABS used at the three T-EBSCG-affiliated hospitals have been described previously [35], and the data reported in the current analysis also include the patient data reported in the earlier publication. Briefly, after pre-operative marking, the patient was placed in the supine position and the arm was abducted 90° to avoid disturbing the operative procedure. Endoscopic video monitors (Olympus Optical Co., Tokyo, Japan) were set up on both sides of the patient’s head and watched by 2 surgeons. An oblique-ended ridged endoscope measuring 5mm in diameter with a viewing angle of 30° was used in all procedures.

In patients for whom SLNB was indicated, a small amount (2–3 mCi) of radioisotope Tc99m was injected intradermally at the site of the tumor before operation. The tumor was located by intra-operative ultrasonography or by palpation and the overlying skin was carefully marked. After induction of general anesthesia, 3 ml of 1% methylene blue (Merck, Darmstadt, Germany) was injected into the breast parenchyma in equally divided aliquots at 5 positions surrounding the hemisphere of the tumor facing the ipsilateral axilla. The breast tissue from the tumor to the axilla was then gently massaged for 5 to 10 minutes. Within 20 to 30 minutes after injection of the blue dye, a handheld gamma probe (Navigator; USSC, Norwalk, CT) was used to identify hot spots and the location of the hottest nodes was marked. An approximately 3-cm oblique axillary incision was then made close to the marks of the hottest nodes and the SLNB was performed.

After the SLNB, the dissection was carried out to the lateral border of the pectoralis major muscle. If the SLN tested positive, a complete ALND with removal of level-I and II lymph nodes was performed. The margin between the pectoralis muscle and breast parenchyma was clearly identified. An endoscopic Ultra Retractor (Johnson & Johnson KK by NCKUH, Karl Storz by CCH, and TMUH) vein harvester was used for the dissection of pectoral muscle fascia and the inferior part of the breast parenchyma. The penetrating vessels were coagulated and cut with bipolar scissors (PowerStar, Johnson & Johnson KK) or a harmonic scalpel to ensure a clear visual field and to maintain hemostasis. The surrounding tissue was pulled up with the Ultra Retractor under endoscopic guidance to create a sufficient working space and a suction tube was used to evacuate mist and smoke.

After the completion of the SLNB, a semi-circular periareolar skin incision or a single axillary incision was made as previously described [23, 24, 33]. A physiological saline solution containing lidocaine 0.05% and epinephrine 1:1,000,000 was injected subcutaneously into the whole breast to minimize bleeding. An approximately 3–5 mm thick skin flap was created using an optical bladeless trocar Xcel (Johnson & Johnson, Tokyo, Japan) under endoscopic guidance. The septa between the skin flap and parenchyma were dissected under endoscopic guidance using endoscissors, bipolar scissors, or a harmonic scalpel.

**Fig 1:**
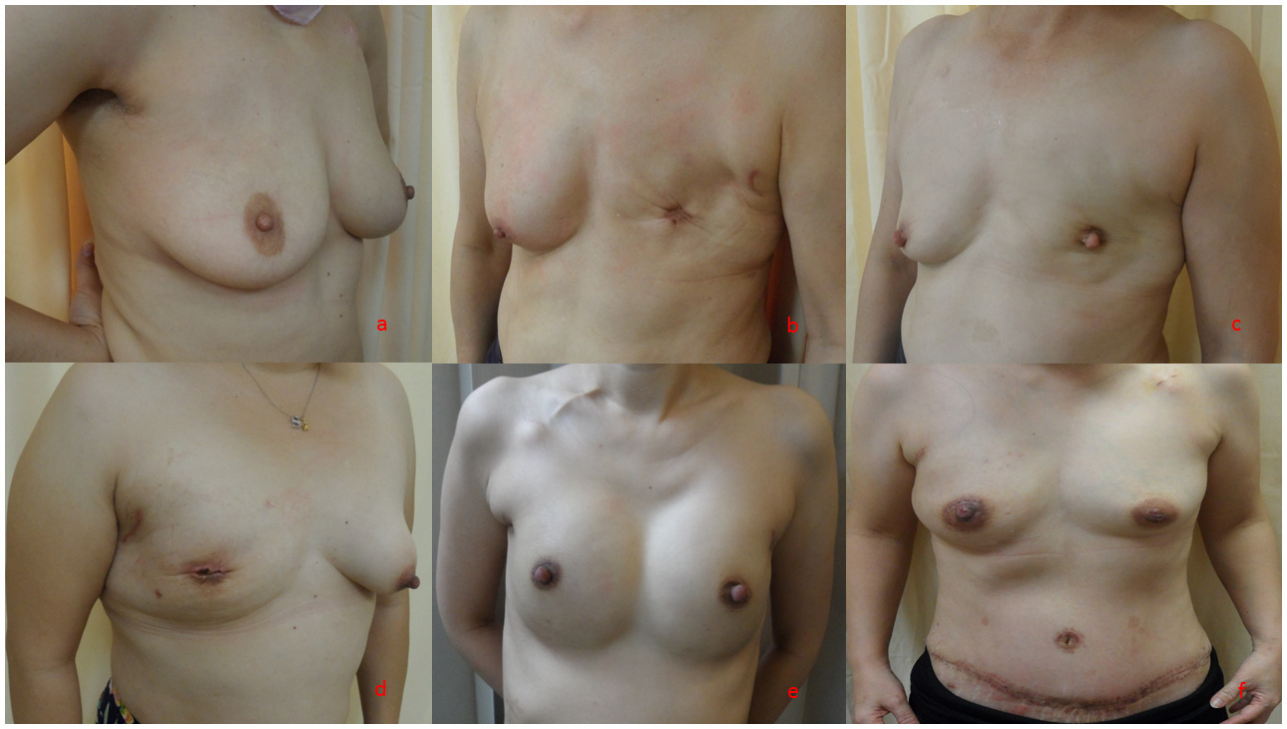
Various types of endoscopy-assisted breast surgery performed for breast cancer. (a) Endoscopic-assisted partial mastectomy (breast conserving surgery), right breast cancer at three-year postoperative follow-up. (b) Endoscopic-assisted skin-sparing mastectomy without reconstruction, left breast cancer at one-year postoperative follow-up. (c) Endoscopic-assisted nipple-sparing mastectomy without reconstruction, left breast cancer at two-year postoperative follow-up. (d) Endoscopic-assisted skin-sparing mastectomy with immediate breast reconstruction with cohesive gel implant, right breast cancer at four-month postoperative follow-up. (e) Endoscopic-assisted nipple mastectomy with immediate breast reconstruction with cohesive gel implant, left breast cancer and right phyllodes tumor post bilateral endoscopic-assisted nipple-sparing mastectomy with gel implant at eight-month postoperative follow-up. (f) Endoscopic-assisted nipple-sparing mastectomy with immediate breast reconstruction with transverse rectus musculocutaneous (TRAM) flap, right breast cancer at six-month postoperative follow-up.

In BCS, video-assisted partial mastectomy was performed through the axillary SLNB incision and an endoscopic Ultra Retractor was used to separate the breast gland and pectoral major muscle. Another periareolar wound was created to separate the breast gland and skin flap if needed, and the specimen was retrieved via the periareolar or axillary incision wound. Then oncoplastic volume displacement technique was performed by moving breast glandular tissue and approximation to prevent parenchyma defect as previously described [5, 24] (Fig 1).

When mastectomy was indicated, mastectomy was performed through an axillary and/or periareolar approach. During NSM, a sub-nipple biopsy specimen was taken from under the nipple areolar complex (NAC) and the intra-operative frozen section was analyzed. If cancer cell invasion was found in the sub-areolar area, the entire NAC was removed, and SSM was performed instead of NSM (Fig 1). The entire breast specimen was removed through the periareolar or axillary wound.

Breast reconstruction was performed immediately or at a later time depending on the patients’ desire for breast reconstruction. Breast reconstructions after EATM were performed using an implant (cohesive Gel implants or tissue expander)[31, 33, 37] or autologous tissue with a latissimus dorsi (LD) flap [34] or a pedicled transverse abdominal musculocutaneous (TRAM) flap as needed [35] (Fig 1).

### Statistical analyses

Differences in continuous variables were tested by the independent *t*-test and are reported as means ± standard deviation (SD). The chi-square test was used for categorical comparisons of data when appropriate. A *p* value of less than 0.05 was considered to indicate statistical significance; all tests were two-tailed. All statistical analyses were performed with the statistical package SPSS (Version 19.0, SPSS, Chicago).

## Results

During the study period, a total of 315 EABS procedures were performed in 292 female patients with breast cancer, including 23 (7.8%) patients with bilateral disease. The mean size of tumors encountered during the 315 EABS procedures was 2.2 ± 1.8 cm (0.1 to 8.5 cm) and 44 (13.9%) of those tumors were multifocal/multicentric. Lymph node metastasis was found during 23.3% of the procedures. Of the 315 EABS procedures conducted during the study period, the majority were performed for pathologic stage II cancer (n = 103, 34.4%), followed by stage I cancer (n = 92, 30.7%), DCIS (stage 0) (n = 86, 28.7%), and stage III breast cancer (n = 19, 6.3%). The demographic and clinical characteristics associated with the 315 EABS procedures are summarized in Table 1.

**Table 1:** Demographic and clinical characteristics of patients who underwent endoscopic-assisted breast surgery.

	N = 292 patients, total 315 EABS
Gender (Female)	292 (100%)
Age (year, mean)	48.1 ± 10.0 (23–80)
Right/Left	150(47.6%)/165(52.4%) (bilateral 23)
Unilateral/bilateral	292(92.2%)/23(7.8%)
Tumor size (invasive, cm)	2.2 ± 1.8 (0.1 to 8.5 cm)
Multifocal/multicentric breast cancer	44/315 (13.9%)
Lymph node (positive/total)	70/300 (23.3%), NA = 15
Clinical stage	N = 273 (NA = 42)
DCIS	66 (24.2%)
Stage I	88 (32.2%)
Stage II	117 (42.9%)
Stage III	2 (0.7%)
Pathologic stage	N = 300 (NA = 15)
DCIS	86 (28.7%)
Stage I	92 (30.7%)
Stage IIa	74 (24.7%)
Stage IIb	29 (9.7%)
Stage IIIa	18 (6%)
Stage IIIc	1 (0.3%)
Mastectomy type	N = 315
Endoscopy assisted NSM	199 (63.2%)
Endoscopy assisted SSM	70 (22.2%)
Endoscopy assisted PM	46 (14.6%)
Axillary surgery	N = 306 (NA = 9)
SLNB (only)	200 (65.4%)
SLNB then ALND	43 (14.1%)
ALND	43 (14.1%)
Not down	20 (6.5%)
Grade	N = 264 (NA = 51)
I	66 (25.0%)
II	127 (48.1%)
III	71 (26.9%)
ER	N = 295 (NA = 20)
Negative	61 (20.7%)
Positive	234 (79.3%)
PR	N = 295 (NA = 20)
Negative	101 (34.2%)
Positive	194 (65.8%)
HER-2	N = 291 (NA = 24)
Negative	242 (83.2%)
Overexpressed	49 (16.8%)
Hormone therapy	168/275 (61.1%) (NA = 40)
Chemotherapy	132/275 (48%) (NA = 40)
Radiotherapy	70/280 (25%) (NA = 35)

EATM: endoscopic assisted total mastectomy, TRAM: transverse abdominal musculocutaneous flap, DCIS: ductal carcinoma in situ, NSM: nipple sparing mastectomy, SSM: skin sparing mastectomy, PM: partial mastectomy, SLNB: sentinel lymph node biopsy, ALND: axillary lymph node dissection. NA: not available. ER: estrogen receptor, PR: progesterone receptor, HER-2: human epidermal growth receptor-2.

**Fig 2:**
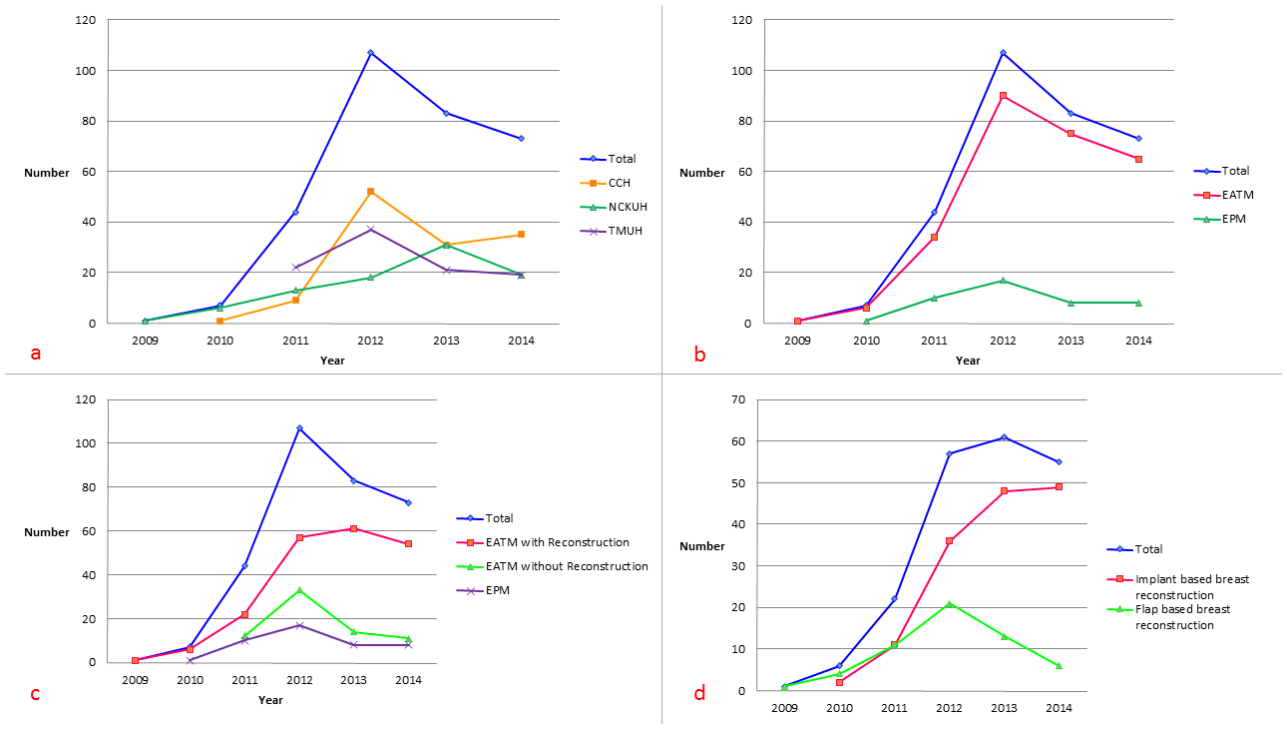
Trend in usage of endoscopy-assisted breast surgery during the period 2009 to 2014 in Taiwan. (a) The number of breast cancer patients who received EABS increased gradually over the past 6 years. The number increased sharply from 2009 to 2012 and then decreased and became stable during the period 2012–2014. This decrease was consistently observed at the three EABS centers in Taiwan. (b) Over the past 6 years (2009–2014), there has been a trend toward use of EABS in the management of breast cancer when total mastectomy was indicated (EATM. (c) Initially E-NSM was performed in conjunction with breast reconstruction. Then EATM without reconstruction was performed gradually. During the study period, there was an increase in the number of EATM procedures performed with IBR, followed by EATM alone without reconstruction and then EPM. (d) The use of gel implants for breast reconstruction increased more rapidly than TRAM flap. Endoscopy-assisted nipple-sparing mastectomy with gel implant reconstruction was the most frequent type of EABS performed at the end of the study.

As seen in Fig 2, the number of breast cancer patients who underwent EABS increased initially from 2009 to 2012 and then stabilized during the period 2012–2014. This trend was consistent at the three T-EBSCG-affiliated hospitals in Taiwan. The most commonly performed EABS was endoscopy-assisted total mastectomy (EATM) (n = 269, 85.4%) followed by endoscopy-assisted partial mastectomy (EAPM, Fig 1) (n = 46, 14.6%). Of the 269 EATMs performed during the study period, 70 (26%) were endoscopic-assisted skin-sparing mastectomy (E-SSM, Fig 1) and 199 (74%) were endoscopic-assisted nipple-sparing mastectomy (E-NSM, Fig 1 and Table 2). During the six-year study period, (2009–2014), there was an increasing trend in the performance of EABS for the management of breast cancer when total mastectomy was indicated (EATM) (Fig 2).

**Table 2:** Types of EABS procedures performed in the study and associated characteristics.

	N = 315 EABS
EATM	N = 269
ENSM	n = 199
E-NSM + Gel implant	118(59.3%)
E-NSM + TRAM	45(22.6%)
E-NSM only	36(18.1%)
ESSM	n = 70
E-SSM + Gel implant	21(30%)
E-SSM + TRAM	10(14.3%)
E-SSM + Tissue expander	4(5.7%)
E-SSM only	35(50%)
OP time all (mins)	282 ± 161 (65–1310)
Mean mastectomy time	219 ± 85 (60–540)
Mean reconstruction time	154 ± 138 (35–770)
Blood loss (ml)	104.5 ± 74.9 (20–650)
Mean mastectomy weight (g)	313.5 ± 147.9 (89–745)
Reconstruction flap weight (g)	500 ± 65.9 (370–600)
Reconstruction implant volume (ml)	287.9 ± 95.0 (120–600)
Hospital stay (days)	5.6 ± 2.1 (2–15)
EPM	N = 46
Mean operation time (mins)	193 ± 69 (65–325)
Mean blood loss (ml)	40.2 ± 20.2 (10–100)
Mean resection partial mastectomy weight (g)	61.3 ± 27.4 (25–128)
Mean hospital stay (days)	3.7 ± 1.1 (2–6)

EABS: endoscopic assisted breast surgery, EATM: endoscopic assisted total mastectomy (including endoscopic assisted nipple sparing mastectomy (E-NSM) and endoscopic assisted skin sparing mastectomy (E-SSM)), TRAM: transverse abdominal musculocutaneous flap, EPM: endoscopic assisted partial mastectomy.

Of the 269 patients who underwent EATM, 198 (73.6%) received IBR. The majority (72.2%, 143/198) of them received implant-based (cohesive Gel implant or tissue expander) reconstruction (Fig 1) and the remaining 27.8% (55/198) received autologous pedicled TRAM flap for breast reconstruction (Fig 1 and Table 2). As seen in Fig 2, we could observe that initially E-NSM was designed to use with breast reconstruction. Then some patients who did not receive breast reconstruction also chose to perform E-NSM gradually. The increasing ratio of EATM with IBR was observed during recent two years, followed by EATM alone without reconstruction and then EPM (Fig 2). Among the methods of breast reconstruction used, EATM and IBR with Gel implant increased more rapidly than TRAM flap (Fig 2). E-NSM with Gel implant reconstruction was the most frequently performed EABS now.

Data on mean operative time, blood loss, and hospital stay associated with the 315 EABS procedures are summarized in Table 2. The wide range in operative time was due to the different types of mastectomies (E-NSM or E-SSM) performed and reconstruction methods used (non-reconstruction, implant or autologous flap). Compared with conventional breast surgeries, EABS did prolong the operation time (Table 3).

**Table 3:** Comparison of operation time between different EABS and conventional operations.

OP time			
Total mastectomy	EATM (n = 269)	Conventional TM (n = 316)	P value
TM only	223.4 ± 72.0 (65–390)	145.5 ± 45.6 (55–605)	<0.01
TM + Gel-implant	282.1 ± 113.4 (110–580)	225.2 ± 75.0 (84–407)	0.0262
TM + TRAM flap	693.2 ± 291.0 (195–1310)	532.7 ± 33.3 (440–720)	0.397
TM + Tissue expander	235.5 ± 127.1 (70–450)	267.5 ± 58.5 (260–270)	0.895
Partial mastectomy	EPM (n = 46)	PM (n = 322)	P value
	193.4 ± 69.3 (65–325)	113.3 ± 45.6 (55–555)	<0.01

EABS: endoscopic assisted breast surgery, EATM: endoscopic assisted total mastectomy (including endoscopic assisted nipple sparing mastectomy (E-NSM) and endoscopic assisted skin sparing mastectomy (E-SSM)), TRAM: transverse abdominal musculocutaneous flap, EPM: endoscopic assisted partial mastectomy, PM: partial mastectomy, TM: total mastectomy.

The complications are listed in Table 4. Overall, the rate of complications associated with EABS was 15.2% and all were minor and wound-related. There were no major or life-threatening complications.

**Table 4:** Complications associated with EABS.

Complications	N = 315
Overall*	**15.2% (48/315)**
Delayed healing of the areolar wound	4.8% (15/315)
Partial ischemia of the nipple-areolar complex #	8.5% (17/199)
Complete necrosis of the nipple-areolar complex#	4% (8/199)
Seroma formation requiring repeat aspiration	2.5% (8/315)
Hematoma formation	1.6% (5/315)
Infection-related complication	1% (3/315)
Breast skin flap ischemia/necrosis	2.5% (8/315)
Implant loss	2.1% (3/143)
TRAM flap partial fat necrosis	9.1% (5/55)
Total TRAM Flap loss	0% (0/55)
Poor wound healing or dehiscence at the donor site	7.3% (4/55)
Abdominal bulging/hernia	0% (0/55)

EABS: endoscopic-assisted breast surgery.

Complications calculation:

*Overall: patients with any one complication were included in the calculation. Each patient could have more than one complication.

^#^ among those who received endoscopic-assisted nipple sparing mastectomy. EATM: endoscopic-assisted total mastectomy, TRAM: transverse abdominal musculocutaneous flap.

The overall positive surgical margin rate was 1.9%, and the positive margin rates associated with EPM and EATM were 6.5% (3/46) and 1.1% (3/269), respectively (Table 5). Postoperative adjuvant hormone therapy, chemotherapy and radiotherapy were given to patients according to current breast cancer guidelines [41, 42] and the results are shown in Table 1. During a median follow-up of 26.8 months (range, 3.3–68.6 months), there were 3 (1%) cases of locoregional recurrence (ipsilateral breast (n = 1), axillary nodes (n = 1), and core needle biopsy tract (n = 1)), 1 (0.3%) case of distant metastasis, and 1 death. The treatment details of these patients are summarized in Table 5. The preliminary outcome of the EABS program in Taiwan is comparable with EABS outcomes reported previously (Table 6).

**Table 5:** Oncologic safety analysis of patients received EABS.

Margin involvement	
**Overall**	6/315(1.9%)
**EATM**	3/269(1.1%)
**E-NSM**	2/199(1%)
A 37 y/o female, pT1cN0M0, post E-NSM + Gel implant reconstruction and superficial margin involvement. Further surgery showed no residual cancer and no recurrence 2.8 years after surgery	
A 42 y/o female, pT1bN0M0, post E-NSM + Gel implant reconstruction with deep margin involvement. No further surgery was performed, and no recurrence was found 3 year post operation.	
**E-SSM**	1/70(1.4%)
A 49 y/o female with left DCIS post E-SSM, margins positive over anterior lateral aspect, further surgery showed no residual cancer	
**EPM**	3/46 (6.5%)
A 54 y/o female with left DCIS post partial mastectomy with lateral margin involvement S/P further wide excision: pathology: residual DCIS. Received radiotherapy without local recurrence 4 years after operation.	
A 40 y/o female, right IDC, pT2N1M0, post EPM + axillary lymph node dissection, pathology: deep margin involvement, no further surgery, received radiotherapy and follow up, no recurrence post 3.5 years	
A 52 y/o female, cT2N1M0, post neoadjuvant chemotherapy, S/P EPM, Margins DCIS (+) lateral; IDC 1mm from lateral and superior margin. S/P further wide excision, pathology: residual cancer.	
**Local regional recurrence**	**(3/315)**
A 47 y/o female, right breast cancer, multifoci, S/P E-NSM, local regional recurrence over the breast 26.5 months later post surgery, S/P further wide excision + axillary lymph node dissection. Currently under letrozole treatment without recurrence.	
A 51 y/o female, right breast cancer S/P E-NSM + Gel implant reconstruction, sentinel lymph node negative, pT1N0M0, ER(low positive), PR(negative) HER-2 positive breast cancer. Refused chemotherapy and herceptin treatment, only received letrozole treatment. Axillary lymph node recurrence 2 years after surgery.	
A 33 y/o female, right breast cancer, pT1bN0M0, S/P E-SSM + TRAM reconstruction, CNB tract recurrence 1 month post surgery, S/P further surgery, no local recurrence after 4.2 years of follow-up	
**Distant metastasis**	**1/292 (0.3%)**
A 33 y/o female with bilateral triple negative breast cancer, right pT2N1Mx, and left pT1N0Mx, received bilateral E-NSM + TRAM reconstruction. Post operation, adjuvant chemotherapy with 4 cycles of FEC (5-FU, epirubicin and cyclophopshamide), and 12 weekly paclitaxel were performed. Post mastectomy radiotherapy for right breast was also delivered due to positive axillary lymph node. She developed brain metastasis 8 months after the operation.	
**Mortality**	**1/292 (0.3%)**
A 33 y/o female with bilateral triple negative breast cancer, received bilateral E-NSM + TRAM reconstruction, developed brain metastasis 8 months post operation. Whole brain irradiation and cisplatin were given, this patient died 6 months later due to brain metastasis.	

EATM: endoscopic assisted total mastectomy, E-NSM: endoscopic assisted nipple sparing mastectomy, E-SSM: endoscopic assisted skin sparing mastectomy, EPM: endoscopic assisted partial mastectomy, DCIS: ductal carcinoma in situ, IDC: infiltrating ductal carcinoma, TRAM: transverse abdominal musculocutaneous flap.

**Table 6:** Oncologic safety of EABS as reported in the literature and in the current study.

**Author**	**year**	**Journal**	**Number**	**OP method**	**margin positive**	**Follow-up(m)**		**Local recurrence**	**Distant metastasis**	**Death**	
Tamoki^21^	2001	Surg Laparosc Endosc Percutan Tech	6	E-PM	0%		one margin+ & convert TM				
Lee^22^	2006	World J Surg	20	E-PM	10%(2/20)		Cosmetic f/u 3m				
Yamashita^19^	2006	J Nippon Med Sch	82	E-PM	0%	25		0%	0%		
Yamashita^38^	2008	Am J Surg	20	E-PM	0%	12		0%	0%		
Nakajima^24^	2009	Ann Surg	551	E-PM	20.5%(113/551)	35		4.2%(23/551)	4.5%(25/551)	1.3%(7/551)	
Park^27^	2011	J Breast Cancer	40	E-PM	5%(2/40)	12		0%			
			681	BCS	10.6%(85/681)	12		0.3%(2/681)			
Ozaki^28^	2013	J Laparoendosc Adv Surg Tech	73	E-PM	1.4%(1/73)	18.1(12–30)		0%			
			90	BCS		43.7(14–70)		1.1%(1/90)			
Takahashi^29^	2014	Surg Today	100	E-PM	4%	23(9–40)		0%	0%	0%	
			150	BCS	3.3%			0%	0%	0%	
T-EBSCG	2016	Current study	46	E-PM	6.5%(3/46)	26.8 (3.3–68.6)		0%	0%	0%	
**Author**	**year**	**Journal**	**Number**	**OP method**	**Reconstruction**	**Margin positive**	**Nipple ischemia**	**Follow-up(m)**	**Local recurrence**	**Death**	**Prothesis loss**
Nakajima^34^	2002	Biomed Pharmacother	17	E-NSM	LDMF	0(0%)		14			
Ho^32^	2002	Surg Endosc	9	E-NSM	prothesis, average 235 ml	0(0%)					
Ito^37^	2008	ANZ J Surg	33	E-NSM	Prothesis, 30/33 (90.9%) average 235 ml	8(24.3%) and excised NAC	3(9.1%) necrosis	51.2 (16–86)	0		9.1%(3/33) infection with prosthesis removed
Fan^30^	2009	Chinese Med J	43	E-NSM	implant	0(0%)	11.6% (5/43)	16.9±11.2 (6–48)	0	0	
Sakamoto^23^	2009	Ann Surg Oncol	87/89	E-NSM	no mention	0%, nipple involved 2(2.2%)	18%(16/89)	52 (16–80)	0		
Tukenmez^33^	2014	J Laparoendosc Adv Surg Tech	10/11	E-NSM	prothesis, implant 4, expander 6	0%,subnipple biopsy 1 (9.1%) positive	0%	3			
**T-EBSCG**	2016	Current study	269	E-NSME-SSM	Prothesis: implant, expander, TRAM	3/269 (1.1%)	12.5% (25/199)	26.8 (3.3–68.6)	1.1 (3/269%)	0.4% (1/269)	Prothesis loss: 2.1%(3/143)

m: months, TM: total mastectomy, f/u: follow-up, E-PM: endoscopic assisted partial mastectomy, E-NSM: endoscopic assisted nipple sparing mastectomy, E-SSM: endoscopic assisted skin sparing mastectomy, BCS: breast conserving surgery, LDMF: latissimus dorsi myocutaneous flap, TRAM: transverse rectus musculocutaneous flap.

## Discussion

Endoscopy is commonly used in the gastrointestinal[11, 12], urologic[13], and thoracic surgical fields [14] but has yet to become a mainstream technique in the field of breast surgery. This is mainly because of the limited working space, the superficial nature of breast lesions, the low morbidity rate and low levels of pain associated with breast surgery[36, 39]. The longer operation time than conventional surgery (Table 3) and the fact that breast tumors can commonly be accessed through small incisions were also the reasons why EABS is not widely performed. Although those are valid reasons for not performing EABS for early stage breast cancer, which can be easily managed with partial breast excision followed by radiotherapy[2, 3], in patients for whom mastectomy is indicated, EABS is an ideal surgery for cosmetic reasons because the wounds required for endoscopic surgery are much smaller than those needed for conventional surgery and can be hidden in inconspicuous locations[23, 36].

The benefits of EABS with regard to incision size were more apparent in EATM than EPM. Of the 315 EABS procedures performed at the three T-EBSCG–affiliated hospitals in Taiwan, the majority (85.4%) were total mastectomies (Table 2). BCS for patients with early stage breast cancer typically does not result in large scars. This might explain why EABS was more frequently performed in the setting of total mastectomy than partial mastectomy over the past six years in Taiwan (Fig 2). However, compared with some oncoplastic breast surgery techniques (e.g., racket incision, batwing incision, and the round block technique), EAPM combined with volume displacement repair results in a smaller scar and better cosmetic outcome [24, 28, 29] (Fig 1).

EATM can be performed through a minimal incision without removing the skin envelope and NAC when there is no evidence of cancer cell invasion[23, 37]. This makes immediate, one-stage breast reconstruction feasible in most circumstances[30, 33]. In our EATM program, E-NSM was feasible in 74% of patients. Breast reconstruction after mastectomy is becoming more common worldwide[43]. We found a similar increase in the use of EATM combined with IBR in our study (Fig 2). Breast reconstruction following EATM normally involves the use of a tissue expander or implants (cohesive Gel or saline)[30–33]. E-NSM or E-SSM combined with IBR involving autologous pedicle TRAM flap is uncommon. In our previous study, we found that EATM with autologous TRAM flap is a safe procedure and that it results in acceptable cosmetic outcome in women with early stage breast cancer[35].

During the six-year study period, we found a marked increase in the number of E-NSM procedures combined with reconstruction with Gel implants (Fig 2). For women with small to medium sized breasts, BCS followed by radiotherapy, in some conditions, may not render a satisfying cosmetic result[44, 45]. E-NSM with IBR (Fig 1) might provide an alternative option for patients because it does not involve radiotherapy and sometimes can result in better cosmetic outcome. This might explain why the number of EPM procedures decreased and the number of E-NSM procedures combined with Gel implant or TRAM flap for reconstruction increased during the study period (Fig 2).

Surgical margin, locoregional recurrence, distant metastasis, and overall survival are the major concerns regarding the oncologic safety of EABS in the management of breast cancer. Previous studies have demonstrated that EABS, either for BCS (E-PM) or total mastectomy (EATM), with or without preservation of the NAC, combined with delayed or immediate breast reconstruction, is associated with good cosmetic results and is oncologically safe [22–24, 27, 30] (Table 6). In our study, at a median follow-up of 26.8 months, the positive surgical margin rate was 1.9%, the locoregional recurrence rate was 1%, and the mortality rate was 0.3%. Similar outcomes have been reported in previous series (Table 6). Nonetheless, larger patient numbers and longer follow-up are needed to establish the oncologic safety of the EABS program in Taiwan.

The preliminary results from the EABS program in Taiwan show that EABS is a safe procedure and results in acceptable cosmetic outcome in women with early stage breast cancer. Our findings together with those from previous studies should help promote this under-used surgical technique in the field of breast cancer.
